# Extended Precordial T Wave Inversions Are Associated with Right Ventricular Enlargement and Poor Prognosis in Pulmonary Hypertension

**DOI:** 10.3390/jcm10102147

**Published:** 2021-05-16

**Authors:** Marcin Waligóra, Matylda Gliniak, Jan Bylica, Paweł Pasieka, Patrycja Łączak, Piotr Podolec, Grzegorz Kopeć

**Affiliations:** 1 Pulmonary Circulation Centre, Department of Cardiac and Vascular Diseases, Faculty of Medicine, Jagiellonian University Medical College, John Paul II Hospital in Krakow, 31-202 Krakow, Poland; marcin.waligora@gmail.com; 2 Students’ Scientific Group of Pulmonary Circulation and Thromboembolic Diseases, Faculty of Medicine, Jagiellonian University Medical College, 31-008 Kraków, Poland; matylda.gliniak@gmail.com (M.G.); janbylica@op.pl (J.B.); ppasieka96@outlook.com (P.P.); patrycja.laczak25@gmail.com (P.Ł.); 3 Department of Cardiac and Vascular Diseases, Faculty of Medicine, Jagiellonian University Medical College, John Paul II Hospital in Krakow, 31-202 Krakow, Poland; ppodolec@interia.pl

**Keywords:** pulmonary hypertension, electrocardiography, T wave inversion, right ventricle dilatation, chronic thromboembolic pulmonary hypertension, pulmonary arterial hypertension

## Abstract

In pulmonary hypertension (PH), T wave inversions (TWI) are typically observed in precordial leads V1–V3 but can also extend further to the left-sided leads. To date, the cause and prognostic significance of this extension have not yet been assessed. Therefore, we aimed to assess the relationship between heart morphology and precordial TWI range, and the role of TWI in monitoring treatment efficacy and predicting survival. We retrospectively analyzed patients with pulmonary arterial hypertension (PAH) and chronic thromboembolic pulmonary hypertension (CTEPH) treated in a reference pulmonary hypertension center. Patients were enrolled if they had a cardiac magnetic resonance (cMR) and 12-lead surface ECG performed at the time of assessment. They were followed from October 2008 until March 2021. We enrolled 77 patients with PAH and 56 patients with inoperable CTEPH. They were followed for a mean of 51 ± 33.5 months, and during this time 47 patients died (35.3%). Precordial TWI in V1–V6 were present in 42 (31.6%) patients, while no precordial TWI were observed only in 9 (6.8%) patients. The precordial TWI range correlated with markers of PH severity, including right ventricle to left ventricle volume $\frac{RVEDV}{LVEDV}$ (R = 0.76, *p* < 0.0001). The presence of TWI in consecutive leads from V1 to at least V5 predicted severe RV dilatation ($\frac{RVEDV}{LVEDV}$ ≥ 2.3) with a sensitivity of 88.9% and specificity of 84.1% (AUC of 0.90, 95% CI = 0.83–0.94, *p* < 0.0001). Presence of TWI from V1 to at least V5 was also a predictor of mortality in Kaplan–Meier estimation (*p* = 0.02). Presence of TWI from V1 to at least V5 had a specificity of 64.3%, sensitivity of 58.1%, negative predictive value of 75%, and positive predictive value of 45.5% as a mortality predictor. In patients showing a reduction in TWI range of at least one lead after treatment compared with patients without this reduction, we observed a significant improvement in RV-EDV and $\frac{\mathrm{RV}-\mathrm{EDV}}{\mathrm{LV}-\mathrm{EDV}}$. We concluded that the extension of TWI to left-sided precordial leads reflects significant pathological alterations in heart geometry represented by an increase in RV/LV volume and predicts poor survival in patients with PAH and CTEPH. Additionally, we found that analysis of precordial TWI range can be used to monitor the effectiveness of hemodynamic response to treatment of pulmonary hypertension.

## 1. Introduction

In a surface electrocardiogram, the period of cardiac repolarization is represented by flat (isoelectric) ST segments and positive T waves in most leads.

In healthy subjects, the surface ECG signals originate mainly from the predominant left ventricle, and the electrical forces of the right ventricle (RV) are reduced or canceled. Thus, to be seen in ECG the RV dilatation must be severe enough to overcome the canceling effects of the left ventricle. Since V1 is close to the RV, it is the most sensitive to changes induced by RV abnormalities. Therefore, repolarization abnormalities originating from the RV are first present in V1, followed by V2 and V3.

In pulmonary hypertension (PH), precordial T wave inversions (TWI) are typically observed in precordial leads V1–V3 [1], but they can also extend beyond lead V3 to the left-sided leads, including V5 and V6 [2,3]. However, the cause and prognostic significance of this extension have not been systematically assessed.

We hypothesized that the extension of TWI range beyond the right-sided precordial leads is related to RV dilatation which extends to the left part of the chest and dominates the left ventricle (LV). Therefore, the aim of this study was to assess the relationship between heart morphology and precordial TWI range in patients with precapillary pulmonary hypertension. We also assessed whether the extension of TWI to the left-sided precordial leads has prognostic significance and whether it can be useful in the monitoring of treatment efficacy.

## 2. Materials and Methods

### 2.1. Patients

All study participants were recruited consecutively at the Department of Cardiac and Vascular Diseases at the John Paul II Hospital in Krakow, Poland, between October 2008 and December 2019. They were further followed until March 2021. Patients were eligible if they were diagnosed with precapillary PH, namely, pulmonary arterial hypertension (PAH) or chronic thromboembolic pulmonary hypertension (CTEPH). The main exclusion criteria were age < 18 years, contraindication to magnetic resonance imaging (cMR), PH due to congenital heart defect, and the presence of left bundle branch block (LBBB) or right bundle branch block (RBBB) in the resting ECG as these conduction abnormalities affect the polarity and morphology of T waves, irrespective of right ventricular overload. Presence of qR complex in V1 was not considered RBBB [4] and was not an exclusion criterion. Both incident patients (those with de novo diagnosis who had not previously been treated for PAH or CTEPH) and prevalent patients (previously diagnosed with PAH or CTEPH and treated for pulmonary hypertension at the time of study enrollment) were eligible.

### 2.2. Clinical Assessment

Clinical assessment included age, patient’s medical history, measurement of weight and height with calculation of body surface area, arterial blood pressure, plasma concentration of N-terminal pro-B-type natriuretic peptide (NT-proBNP), six-minute walk test, and assessment of the World Health Organization functional class (WHO FC). All data were acquired during a single hospitalization scheduled for diagnostic reasons. Patients were treated with PAH-specific drugs according to European Society of Cardiology (ESC) guidelines and local regulations by the National Health Fund.

### 2.3. Cardiac Catheterization

Right heart catheterization (RHC) was performed in a supine position from the right or left femoral vein or right or left internal jugular vein access using a Swan–Ganz catheter.

Right heart catheterization was performed in awake patients under local anesthesia. All measurements, including acquisition of pressure waves, were made at end expiration. Cardiac output was measured using the Fick oxygen consumption method. Blood oxygen saturation was measured with a Co-oximeter OSM3 (Radiometer, Copenhagen, Denmark), and the oxygen consumption was estimated as 125 mL/min/kg^2^. Pulmonary vascular resistance (PVR) was calculated as the difference between mean pulmonary arterial pressure (mPAP) and pulmonary artery wedge pressure divided by cardiac output.

### 2.4. Cardiovascular Magnetic Resonance Imaging

Breath-hold, ECG-gated imaging was performed according to current standards as described previously [2]. Assessed parameters included left ventricular end-diastolic volume (LV_EDV_), right ventricular end-diastolic volume (RV_EDV_), left ventricular end-systolic volume (LV_EDV_), right ventricular end-systolic volume (RV_EDV_), and right and left myocardial mass and ejection fractions (EF). End-diastolic volume and myocardial mass were indexed to body surface area. RV_EDV_ to LV_EDV_ ratio ($\frac{RVEDV}{LVEDV}$) was calculated. Severe $\frac{RVEDV}{LVEDV}$ was defined as ≥2.3, which was found to be the optimal cut-off value to predict mortality in PAH patients [5].

### 2.5. Electrocardiography

A standard 12-lead surface electrocardiogram (10 mm = 1 mV, 25 mm/s) was acquired in a supine position during quiet respiration. For the purposes of the present study, we assessed precordial T waves’ morphologies (positive, negative) [6]. T wave inversion (TWI) was defined as the presence of a negative T wave of amplitude >0.1 mV [6]. TWI range was defined as the number of consecutive precordial leads with a negative T wave. For example, TWI range was 0 if no negative T waves were present or 3 if negative T waves were present in leads V1, V2, and V3.

### 2.6. Follow-Up

To confirm the association between change in the structure of cardiac chambers and TWI, we compared patients with or without TWI improvement at follow-up examination. For this analysis, we used data of patients who had cMR examination at baseline and at least 3 months after treatment of PAH or CTEPH. TWI improvement was defined as a reversal of negative T waves of at least 1 lead, including at least the most leftward precordial lead with a negative T wave in a given patient. All-cause mortality was ascertained by data collection either (1) from the medical registry of the hospital, (2) from the Department of Nationals’ and Foreigners’ Affairs, or (3) through phone follow-up. The observation period started at the baseline assessment of the patient in our center and extended for at least 12 months until the end of March 2021.

### 2.7. Statistical Analysis

The relationship between precordial TWI range and parameters obtained by cMR or RHC was assessed by Spearman or Pearson correlation test when appropriate. To compare the two groups with and without TWI improvement, we used the Student’s *t*-test or Mann–Whitney *U* test when appropriate for continuous variables and chi-square test for categorical variables. Based on the presence or absence of RV to LV end-diastolic volume ratio ≥ 2.3, patients were subdivided into $\frac{RVEDV}{LVEDV}$ ≥ 2.3 or <2.3. The receiver operating characteristic (ROC) curves with cut-off values of TWI range yielding the maximum sensitivity and specificity for predicting $\frac{RVEDV}{LVEDV}$ ≥ 2.3 were generated. The area under the ROC curve (AUC) was used as a measure of the test accuracy to discriminate between patients with and without $\frac{RVEDV}{LVEDV}$ ≥ 2.3. Kaplan–Meier curves were used for graphic delineation of survival differences between the group with precordial TWI in V1–V4 and the group with TWI in V1–V5 or V1–V6. Logistic regression analysis was used to establish predictors of death. To avoid overfitting of the model, we limited the number of independent variables to 3, namely, TWI range up to V5 or V6, age, and sex. Continuous variables are reported using means and standard deviations, and categorical variables are described as counts and percentages. Statistical analysis was performed with Statistica PL software (TIBCO Software Inc. 2017, version 13, CA, USA) and MedCalc Statistical software version 16.8 (MedCalc software bvba, Ostend, Belgium; 2016). The significance level was set at an alpha level of 0.05. The study protocol conforms to the ethical guidelines of the 1975 Declaration of Helsinki and was approved by the institutional Ethics Committee.

## 3. Results

### 3.1. Characteristics of the Study Sample

Between February 2008 and December 2019, we diagnosed 271 PAH and 107 CTEPH patients. Of the PAH patients, 99 were excluded due to the diagnosis of congenital heart disease, 10 due to inability to hold breath during cMR, 26 due to contraindications for cMR, 43 because of lack of consent for cMR, and 16 due to the presence of bundle branch blocks (RBBB, *n* = 15; LBBB, *n* = 1). Of patients with CTEPH, 10 were excluded due to contraindications for cMR, 22 due to lack of consent for cMR, 5 due to inability to hold breath during cMR, 1 due to rapid AF, and 13 due to the presence of RBBB. Finally, 133 patients met the inclusion criteria (PAH, *n* = 77; CTEPH, *n* = 56). The detailed characteristics of the baseline study group are presented in Table 1.

### 3.2. T Wave Inversions

The inversion of T waves in all precordial leads was present in 42 (31.6%) patients, while no precordial TWI were observed in 9 (6.8%) patients. TWI solely in V1 were present in 27 (20.3%) patients, TWI in V1 and V2 in 12 (9%), TWI in V1 to V3 in 16 (12%), TWI in V1 to V4 in 15 (11.3%), and TWI in V1 to V5 in 12 (9%) patients.

**Figure 1:**
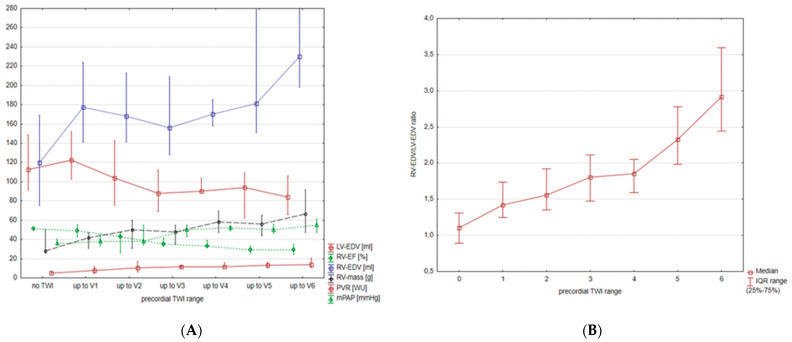
(**A**) Right ventricle ejection fraction (RV_EF_), right ventricle end-diastolic volume (RV_EDV_), right ventricle mass (RV_m_), mean pulmonary arterial pressure (mPAP), and pulmonary vascular resistance (PVR) in relation to the presence of T wave inversions (TWI) in consecutive precordial leads. Data are presented as median and interquartile range. Each unit on the y-axis corresponds to one unit for the presented variables (e.g., 1 for 1 mL of volume or for 1% of ejection fraction, etc.). (**B**) RV_EDV_ to left ventricle end-diastolic volume ratio ($\frac{RVEDV}{LVEDV}$ ratio) in relation to the presence of T wave inversions (TWI) in consecutive precordial leads. Data are presented as median and interquartile range.

**Figure 2:**
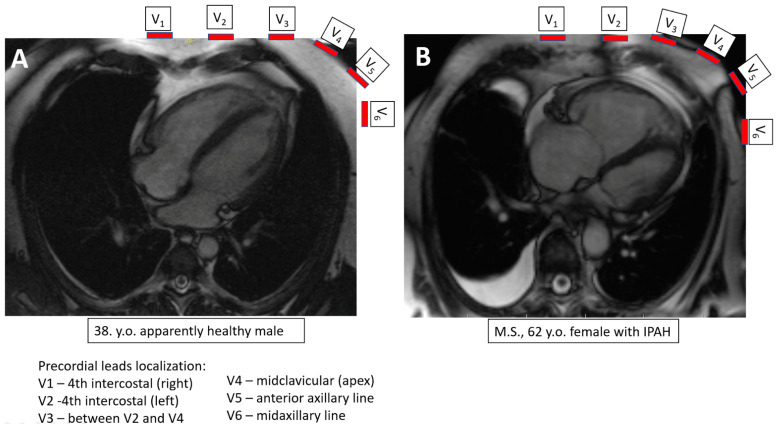
Localization of precordial leads in relation to the left and right ventricles in an apparently healthy subject (**A**) and in a patient with advanced pulmonary hypertension. y.o.–years old (**B**). The left-sided leads (V4–V6) in a patient with advanced pulmonary arterial hypertension “look” at the right ventricle instead of the left ventricle. IPAH–idiopathic pulmonary arterial hypertension.

**Figure 3:**
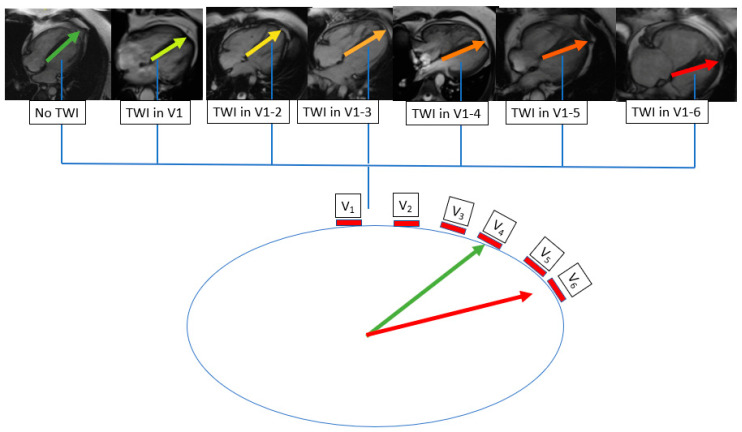
Graphical representation of right ventricle dislocation towards lead V6 in patients with different stages of pulmonary hypertension. In a healthy subject, lead V4 is located over the apex. As the right ventricle volume increases and the left ventricle volume decreases, the apex is rotated towards lead V6. Consequently, most precordial leads represent electrical processes in the right ventricle rather than in the left ventricle. TWI–T waves inversions.

**Figure 4:**
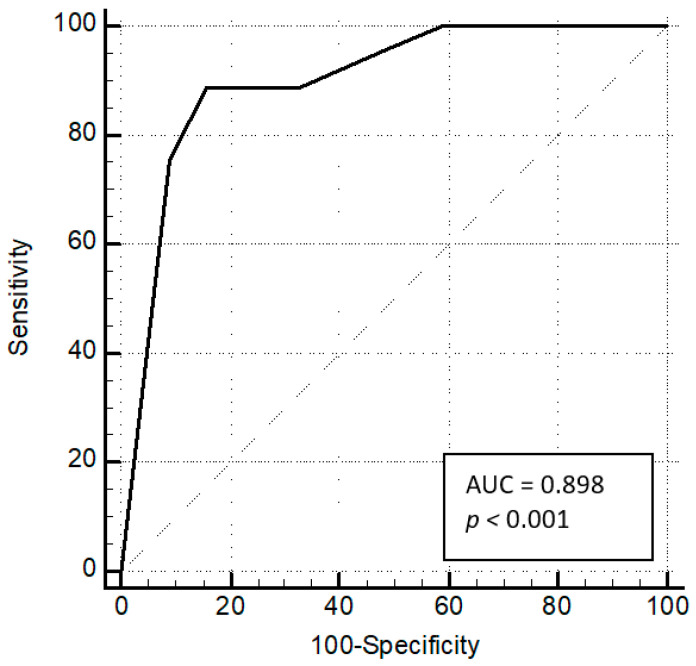
Precordial T wave inversion (TWI) range as a predictor of severe right ventricular dilatation (right ventricular end-diastolic volume/left ventricular end-diastolic volume > 2.3). Area under curve of 0.90, 95% CI = 0.83–0.94, *p* < 0.0001.

The TWI range correlated with markers of PH severity as presented in Table 2. In Figure 1A,B we present markers of advanced PAH in relation to the TWI range. The strongest correlation was found between precordial TWI range and $\frac{RVEDV}{LVEDV}$ (R = 0.76, *p* < 0.0001, Table 2, Figure 1B). The graphical interpretation of increasing TWI range alongside increasing $\frac{RVEDV}{LVEDV}$ is presented in Figure 2 and Figure 3. The cut-off value of TWI yielding the maximum sensitivity and specificity for predicting $\frac{RVEDV}{LVEDV}$ ≥ 2.3 was 5, with a sensitivity of 88.9% and specificity of 84.1% (AUC of 0.90, 95% CI = 0.83–0.94, *p* < 0.0001, Figure 4).

### 3.3. Comparison of Patients with and without TWI Range Improvement in Follow-Up

Thirty-eight patients were identified to have a follow-up cMR. Patients with no improvement in the precordial TWI range were diagnosed with IPAH (*n* = 11), PAH associated with connective tissue disease (CTD-PAH, *n* = 1), or CTEPH (*n* = 8), while patients with improvement in the precordial TWI range were mostly CTEPH (*n* = 12) followed by CTD-PAH (*n* = 4) and IPAH (*n* = 2). Patients who had improvement in precordial TWI were treated with balloon pulmonary angioplasty (*n* = 8) or pulmonary endarterectomy (PEA, *n* = 1), followed by sildenafil (*n* = 7), treprostinil (*n* = 1), and calcium channel blocker (CCB, *n* = 1). Patients without improvement were treated with BPA (*n* = 5), supportive therapy only (inoperable CTEPH in era before BPA, *n* = 3), CCB (*n* = 1), iloprost (*n* = 2), sildenafil (*n* = 7), ERA (*n* = 1), and PEA (*n* = 1). Detailed characteristics are presented in Table 3.

After a mean time between two consecutive cMRs of 11.2 ± 6.7 months, we observed that patient who improved in TWI range as compared with the patients who did not improve had greater decrease in NT-proBNP level, RV_EDV_ and $\frac{RVEDV}{LVEDV}$ ratio (Table 3). Additionally, a change in RV-EDV and $\frac{RVEDV}{LVEDV}$ correlated with a change in precordial TWI range in patients with regression in precordial TWI range of at least one lead (R = 0.65, *p* = 0.006 and R = 0.71, *p* = 0.002, respectively).

### 3.4. Follow-Up

**Figure 5:**
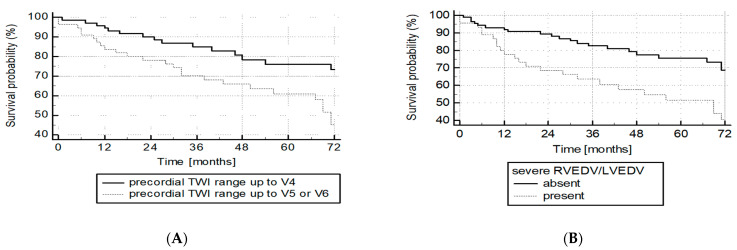
Survival differences between groups with TWI ranging up to V4 or extending to V5/V6 (**A**), *p* = 0.02. Survival differences between groups with severe $\frac{RVEDV}{LVEDV}$ dilatation ($\frac{RVEDV}{LVEDV}$ ≥ 2.3 ≥ 2.3) or with $\frac{RVEDV}{LVEDV}$ < 2.3 (**B**), *p* = 0.008.

During a mean follow-up of 51 ± 33.5 months, 47 patients died (35.3%). Additionally, two patients were lost to follow-up (both diagnosed in years 2008–2009). The presence of a TWI range of 5 or more at baseline ECG was a significant predictor of mortality (Figure 5A). This predictive value was also confirmed for the severe right ventricle dilatation ( $\frac{RVEDV}{LVEDV}\geq 2.3$ ) as measured in cMR and showed in Figure 5B. In the multivariable logistic regression analysis including age, sex, and TWI range, we found that only TWI range of 5 or 6 predicted death: odds ratio = 2.47 (95% CI 1.16–5.3, *p* = 0.02). Neither age (OR = 1.02, 95% CI: 0.99–1.04, *p* = 0.22) nor male sex (OR = 1.13, 95% CI: 0.52–2.5, *p* = 0.75) predicted survival in our population.

## 4. Discussion

In the current study, we showed that most patients with pulmonary arterial hypertension or chronic thromboembolic pulmonary hypertension were characterized by T wave inversion in one or more precordial leads of the resting ECG. The TWI range correlated with markers of pulmonary hypertension severity and reflected the overload of the RV defined by an increased RV to LV ratio and an increased NT-proBNP level. It was also a predictor of mortality. Additionally, we showed that a decrease in TWI range in response to targeted therapies reflected an improvement in right ventricular dominance and RV overload.

In pulmonary hypertension, the presence of negative T waves in precordial leads is frequent [3] yet not well understood. In a study by Bonderman et al. [7], the presence of RV strain in ECG (defined as the presence of ST segment deviation and T wave inversion in leads V1–V3) was observed frequently in the precapillary PH group (78.6%) and relatively rarely in postcapillary PH (7.8%). Based on the results, the inclusion of RV strain in models predicting the diagnosis of precapillary pulmonary hypertension was proposed. When RV strain was used in combination with NT-proBNP, it predicted precapillary pulmonary vascular disease with a sensitivity of 100% and specificity of 19.3%, which highlights the importance of assessing repolarization abnormalities in patients suspected of having PH.

To date, the mechanisms and the prognostic role of TWI have not been analyzed in patients with RV overload as opposed to studies on the left ventricle. In patients with LV overload due to systemic hypertension, TWI were attributed to subendocardial ischemia which was present despite the absence of coronary artery disease [8]. In these studies, the regression of TWI was associated with lower cardiovascular morbidity and mortality [9]. The reversal of TWI was also described in patients with aortic stenosis after valve replacement surgery [10].

Some recent studies showed that unloading of the right ventricle in pulmonary hypertension can also improve several ECG parameters. In a study by Pilka et al., invasive treatment of CTEPH with balloon pulmonary angioplasty (BPA) was associated with a decrease in PVR and in mPAP, which was reflected by ECG improvement including a decrease in the frequency of negative T waves in leads V1–V3 from 55% before BPA to 22% after finishing BPA treatment [11,12]. Similar observations were found in a study by Nishiyama et al. where negative T waves in V1–V3 were observed in 56% of patients before BPA and only in 8.3% after completing BPA treatment [13].

Still, the knowledge on the role of TWI in left-sided precordial leads in pulmonary hypertension is scarce. Sato et al. observed a decrease in ECG strain (defined as down-sloping convex ST segment with an inverted asymmetrical T wave opposite to the QRS axis) in leads V2 (from 52% to 30%), V3 (from 45% to 25%), and V4 (from 34% to 25%) in patients treated with intravenous epoprostenol, but these changes were not associated with better survival [14]. In another study, serial ECG tracings were collected at the time of PAH diagnosis and before the patient’s death. It was observed that the frequencies of negative T waves in V1–V3 were similar at both timepoints [15]. However, the frequency of negative T waves in the inferior leads increased from 30.8% to 60%. In our study, the presence of negative T waves in the left-sided precordial leads appeared to predict mortality in PH patients.

The adaptation of the RV is crucial in PH. Its maladaptation, enlargement, and failure are associated with advanced PH and poor prognosis [16,17,18,19,20]. The new finding of our study is that TWI range is proportional to the maladaptive response of the RV and that the presence of negative T waves in left-sided precordial leads reflects a very high-risk enlargement of the RV defined as an RV to LV volume ratio above or equal to 2.3 [5]. It has been previously described that the threshold of RV/LV volume ≥2.3 was associated with poor prognosis in PAH patients. We have also shown that regression of T wave inversions in precordial leads is a sensitive marker of improvement in the RV and LV volumes. This suggests that a decrease in RV overload may be monitored by changes in TWI range in surface ECG. Interestingly, this ECG change did not transfer directly to an improvement in WHO functional class or 6 MWD. This might result from the multifactorial nature of exercise capacity but also from the high sensitivity of TWI range to a decrease in the RV/LV volume ratio.

The main strength of our study is its novelty. We have shown for the first time the role of TWI in left-sided precordial leads in the prognosis of PH patients and in the monitoring of treatment efficacy. Second, we enrolled a large cohort of PAH and CTEPH patients despite the rarity of these conditions [21,22,23]. Third, we described the correlation between TWI and changes in RV and LV morphology in patients with precapillary PH.

Our study also has some limitations. First, the study is single-center and retrospective. Second, the timespan of study group enrollment was long and during this time the treatment approach for patients with PH improved, which could affect the outcome.

## 5. Conclusions

The extension of TWI to left-sided precordial leads reflects a significant increase in RV/LV volume ratio and predicts poor survival in patients with PAH and CTEPH. Additionally, analysis of the precordial TWI range can be used to monitor the effectiveness of hemodynamic response to treatment of pulmonary hypertension.

## Figures and Tables

**Table 1:** Characteristics of study patients at the time of study enrollment. Abbreviations (in order of appearance): PAH, pulmonary arterial hypertension; IPAH, idiopathic pulmonary arterial hypertension; CTD-APAH, pulmonary arterial hypertension associated with connective tissue disease; CTEPH, chronic thromboembolic pulmonary hypertension; CAD, coronary artery disease; PCI, percutaneous coronary intervention; COPD, chronic obstructive pulmonary disease; WHO FC, World Health Organization functional class; NT-proBNP, N-terminal pro-B-type natriuretic peptide; 6 MWD, distance in 6-min walking test; RHC, right heart catheterization; iRBBB, incomplete right bundle branch block; RBBB, complete right bundle branch block; LBBB, left bundle branch block; mPAP, mean pulmonary artery pressure; RAP, right atrial pressure; CO, cardiac output; CI, cardiac index; SpO_2_, oxygen saturation; MVB, mixed venous blood; PVR, pulmonary vascular resistance; LV-m, left ventricle mass; LV-EDV, left ventricle end-diastolic volume; LV-EF, left ventricle ejection fraction; RV-EDV, right ventricle end-diastolic volume; RV-m, right ventricle mass; RV-EF, right ventricle ejection fraction; RAA, right atrial area.

*n*	133
Etiology:	
PAH (*n* (%))	77
IPAH (*n* (%))	64 (48.1%)
CTD-APAH (*n* (%))	13 (9.8%)
CTEPH (*n* (%))	56 (42.1%)
Concomitant diseases:	
CAD with prior PCI (*n* (%))	4 (3%)
Arterial hypertension (*n* (%))	43 (32.3%)
Diabetes (*n* (%))	11 (8.3%)
COPD (*n* (%))	4 (3%)
Thyroid gland disease (*n* (%))	21 (15.8%)
Sex, female (*n* (%))	86 (64.7%)
Age (years)	54.7 ± 15.6
Weight (kg)	72.2 ± 17
WHO FC:	
I	0
II	18 (13.5%)
III	97 (72.9%)
IV	18 (13.5%)
Newly diagnosed (“incident cases”) (*n* (%))	113 (85%)
NT-proBNP (pg/mL)	2539 ± 3167
6 MWD (m)	337.5 ± 117.4
ECG:	
Heart rate (bpm)	77.6 ± 14
RHC:	
mPAP (mmHg)	48.1 ± 14.3
RAP (mmHg)	6.1 ± 4.6
Systolic blood pressure (mmHg)	127 ± 21.3
Diastolic blood pressure (mmHg)	77.8 ± 17.2
CO (L/min)	3.3 ± 1.4
CI (L/min/m^2^)	1.8 ± 0.7
Peripheral blood SpO_2_ (%)	92.2 ± 5.3
MVB SpO_2_ (%)	59.7 ± 9.7
PVR (WU)	12.1 ± 6.1
cMR:	
LV_m_ (g)	96.4 ± 28.7
LV_EDV_ (mL)	105 ± 42.8
LV_EF_ (%)	61.8 ± 9.5
RV_EDV_ (mL)	200 ± 75
RV_m_ (g)	57.3 ± 32.4
RV_EF_ (%)	37.2 ± 11.7
RAA (cm^2^)	30.9 ± 9.4

**Table 2:** Correlation between structural, functional, and biochemical markers of pulmonary hypertension severity and the range of T wave inversions in precordial leads. Abbreviations (in order of appearance): TWI, T wave inversions; cMR, cardiac magnetic resonance imaging; RV_EDV_, right ventricle end-diastolic volume; RV_m_, right ventricle mass; RV_EF_, right ventricle ejection fraction; LV_EDV_, left ventricle end-diastolic volume; LM_m_, left ventricle mass; LV_EF_, left ventricle ejection fraction; $\frac{RVEDV}{LVEDV}$, left ventricle to right ventricle volume ratio; RAA, right atrial area; mPAP, mean pulmonary arterial pressure; PVR, pulmonary vascular resistance; CI, cardiac index; RAP, right atrial pressure; NT-proBNP, N-terminal pro-B-type natriuretic peptide; 6 MWD, six-minute walking test distance.

	R Coefficient for Comparison with Listed Variable and Precordial TWI Range	*p*
Clinical variables		
Age (years)	−0.04	0.69
NT-proBNP (pg/mL)	0.46	<0.0001
6 MWD (m)	−0.37	0.0001
cMR variables		
RV_EDV_ (mL)	0.44	<0.0001
RV_m_ (g)	0.43	<0.0001
$\frac{\mathrm{RVm}}{\mathrm{RVEDV}}$ (g/mL)	0.09	0.34
RV_EF_ (%)	−0.61	<0.0001
LV_EDV_ (mL)	0.35	0.0001
LV_m_ (g)	−0.07	0.4
LV_EF_ (%)	−0.19	0.03
$\frac{RVEDV}{LVEDV}$	0.68	<0.0001
RAA (cm^2^)	0.42	<0.0001
Hemodynamic variables		
mPAP (mmHg)	0.42	<0.0001
PVR (WU)	0.48	<0.0001
CI (L/min/m^2^)	−0.26	0.004
RAP (mmHg)	0.27	0.002

**Table 3:** Comparison of patients with and without improvement in precordial TWI range after specific treatment. Abbreviations (in order of appearance): WHO FC, World Health Organization functional class; NT-proBNP, N-terminal pro-B-type natriuretic peptide; 6 MWD, distance in 6-min walking test; cMR, cardiac resonance imaging; LV_m_, left ventricle mass; LV_EDV_, left ventricle end-diastolic volume; LV_EF_, left ventricle ejection fraction; RV_EDV_, right ventricle end-diastolic volume; RV_m_, right ventricle mass; RV_EF_, right ventricle ejection fraction; RAA, right atrial area; RHC, right heart catheterization; mPAP, mean pulmonary artery pressure; RAP, right atrial pressure; CI, cardiac index; PVR, pulmonary vascular resistance.

	Patients with No Improvement in Precordial TWI Range (*n* = 20)	Patients with Improvement in Precordial TWI Range (*n* = 18)	*p*
Clinical and ECG characteristics			
Baseline WHO FC	3.05 ± 0.22	2.94 ± 0.42	0.33
Δ WHO FC	−0.33 ± 0.7	−0.61 ± 0.85	0.27
Baseline NT-proBNP (pg/mL)	1812 ± 1664	2407 ± 2483	0.4
Δ NT-proBNP (pg/mL)	−714 ± 1112	−2202 ± 2198	0.01
Baseline 6 MWD (m)	358 ± 110	316 ± 81.7	0.21
Δ 6 MWD (m)	+51.4 ± 58.9	+ 77.9 ± 68.2	0.22
cMR			
Baseline RV_EF_ (%)	35.9 ± 12.1	39.2 ± 9.4	0.35
Δ RV_EF_ (%)	+4.8 ± 9.4	+10.2 ± 13.5	0.25
Baseline RV_EDV_ (mL)	188.1 ± 58.2	182 ± 53.1	0.74
Δ RV_EDV_ (mL)	+10.9 ± 41	−40.9 ± 37.4	<0.0001
Baseline RV_m_ (g)	52.8 ± 18.2	50.4 ± 26.9	0.75
Δ RV_m_ (g)	−3.1 ± 19	−15.1 ± 27.8	0.17
Baseline LV_EDV_ (mL)	95.1 ± 35.8	95.2 ± 26.1	0.99
Δ LV_EDV_ (mL)	+2.5 ± 18.3	+29.6 ± 26.6	0.005
$\mathrm{Baseline}\;\frac{RVEDV}{LVEDV}$	2.16 ± 0.87	1.97 ± 0.52	
$\Delta \frac{RVEDV}{LVEDV}$	0 ± 0.55	−0.8 ± 0.46	<0.0001
Baseline RAA (cm^2^)	31.3 ± 13.6	29 ± 13.6	0.53
Δ RAA (cm^2^)	+0.3 ± 5.2	−4.8 ± 7.5	0.05

## Data Availability

The data presented in this study are available on request from the corresponding author.
